# The Prognostic Value of a Single, Randomly Timed Circulating Tumor DNA Measurement in Patients with Metastatic Melanoma

**DOI:** 10.3390/cancers14174158

**Published:** 2022-08-27

**Authors:** Aurelio Boerlin, Elisa Bellini, Patrick Turko, Phil F. Cheng, Mitchell P. Levesque, Reinhard Dummer, Egle Ramelyte

**Affiliations:** 1 Department of Dermatology, University Hospital Zurich, 8091 Zurich, Switzerland; 2 Medical Faculty, University of Zurich, 8006 Zurich, Switzerland

**Keywords:** ctDNA, melanoma, tumor progression, PET-CT, S100, biomarker

## Abstract

**Simple Summary:**

In this study, we investigated the associations of circulating tumor DNA (ctDNA), measured at a random time point during the patient’s treatment, with tumor progression and routine blood markers (protein S100, lactate dehydrogenase (LDH), and C-reactive protein (CRP)) in a cohort of patients with metastatic melanoma. Detectable ctDNA was associated with the presence of extracerebral disease, tumor progression, and poorer overall survival (OS). Elevated S100 and CRP was correlated with detectable ctDNA, whereas LDH was not. Our results further support the use of ctDNA in the clinical management of patients with metastatic melanoma.

**Abstract:**

Melanoma currently lacks validated blood-based biomarkers for monitoring and predicting treatment efficacy. Circulating tumor DNA (ctDNA), originating from tumor cells and detectable in plasma, has emerged as a possible biomarker in patients with metastatic melanoma. In this retrospective, single-center study, we collected 129 plasma samples from 79 patients with stage IIIB–IV melanoma as determined by the American Joint Committee on Cancer (AJCC, 8th edition). For the determination of ctDNA levels, we used eight different assays of droplet digital polymerase chain reaction (ddPCR) to detect the most common hotspot mutations in the *BRAF* and *NRAS* genes. The aim of the study was to investigate the association of the detectability of ctDNA at a non-prespecified time point in a patient’s treatment with tumor progression, and to correlate ctDNA with commonly used biomarkers (protein S100, LDH, and CRP). Patients with detectable ctDNA progressed more frequently in PET-CT within 12 months than those without detectable ctDNA. Detectability of ctDNA was associated with shorter OS in univariate and multivariate analyses. ctDNA was detectable in a statistically significantly larger proportion of patients with distant metastases (79%) than in patients with no distant metastases or only intracranial metastases (32%). Elevated protein S100 and CRP correlated better with detectable ctDNA than LDH. This study supports the potential of ctDNA as a prognostic biomarker in patients with metastatic melanoma. However, additional prospective longitudinal studies with quantitative assessments of ctDNA are necessary to investigate the limitations and strengths of ctDNA as a biomarker.

## 1. Introduction

During tissue remodeling and cell death, DNA is released into the extracellular space. After entry into the bloodstream, cell-free DNA (cfDNA) derived from tumoral tissue is called circulating tumor DNA (ctDNA). Analysis of ctDNA in peripheral blood allows for the detection of tumor-specific genomic variants in various malignancies [1,2,3,4,5,6]. 

ctDNA is increasingly being tested as a liquid biomarker in patients with metastatic melanoma. Plasma ctDNA levels have also been applied in therapeutic clinical trials to monitor treatment effects and predict outcomes [7,8] or in routine diagnostics to monitor relapse following completed systemic therapy or surgery [2,9,10]. Detectable plasma ctDNA levels at baseline were associated with worse overall survival (OS) compared to patients with undetectable ctDNA, which had longer progression-free survival (PFS) and better OS rates [7,8,9].

However, plasma levels show large intra- and interpatient variability due to tumor burden, location, and therapeutic intervention [1,7,11,12,13,14,15,16].

In patients with metastatic melanoma, plasma lactate dehydrogenase (LDH) is an established prognostic blood-derived biomarker [17]. Additionally, protein S100 has been identified as a candidate marker to monitor tumor burden [18,19]. However, these routine blood markers lack sufficient specificity and sensitivity for the prediction of treatment response and prognosis. For the detection of tumor progression, imaging with PET-CT scans is still the recommended method [20]. 

The aims of this project were, first, to investigate whether a single ctDNA measurement, taken from patients at a non-prespecified time point during the clinical course of metastatic melanoma, can be a useful predictor of tumor progression; and second, to analyze the correlations between ctDNA and routine blood markers.

## 2. Materials and Methods

### 2.1. Patients

**Figure 1:**
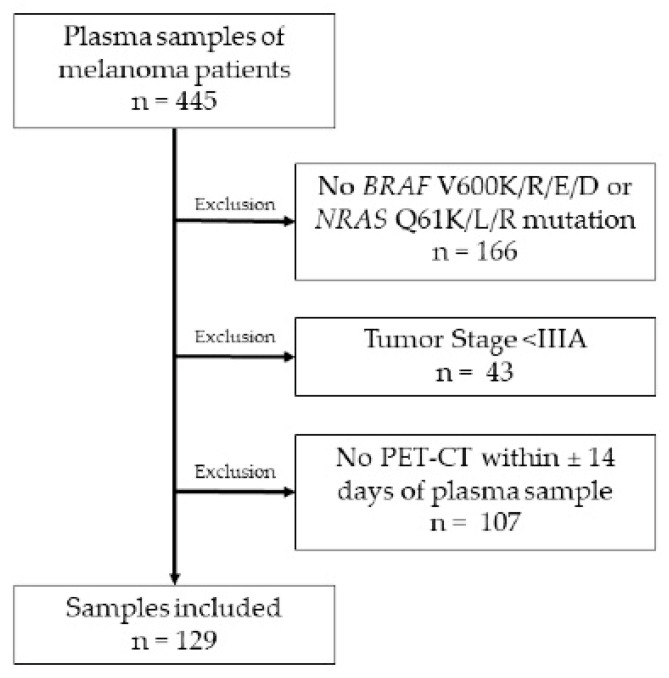
Overview of sample selection.

In this retrospective, single-center study, we analyzed patients with AJCC 8th edition stage IIIB–IV cutaneous melanoma, treated at within the Department of Dermatology at the University Hospital of Zurich. Patients were included or excluded according to the criteria in Figure 1. Tumor mutations were determined using next-generation sequencing [21] during the course of routine diagnostics.

The last treatment before the ctDNA sample collection, and all following treatments up to 12 months after the sample collection, were recorded. Patients were not excluded if they switched treatment modalities within the observation period due to a lack of response or treatment tolerability.

The demographic, clinical, and pathological features of eligible patients, including age, gender, tumor stage, Breslow thickness and ulceration of primary tumor, location of the metastasis, and tumor treatment were obtained from our institutional database.

### 2.2. Routine Blood Markers

Routine blood markers—LDH, protein S100, and CRP values—were analyzed within three days of a blood draw for ctDNA. Markers were considered elevated when above the ULN (upper limit of normal).

### 2.3. ctDNA Assessment

We collected plasma samples of patients at non-predefined time points between October 2015 and March 2021, and only when a blood draw was otherwise necessary. These time points were aligned with scheduled visits as part of routine treatment or during follow-up care and were not specifically related to the onset of therapeutic interventions or tumor progression.

Circulating cfDNA was isolated from 2–5 mL of plasma using the QIAamp circulating nucleic acid kit (Qiagen) for the QIAvac 24 plus vacuum system instrument (Qiagen), according to the manufacturer‘s instructions.

A fixed volume of 5 μL of each cfDNA isolation was mixed in duplicate with droplet digital PCR (ddPCR) multiplex supermix (Bio-Rad) and a primer-probe mix (prototype developed by Oncobit AG) that specifically amplifies and detects a *BRAF* or *NRAS* mutated allele (COSM473, COSM474, COSM475, COSM476, COSM477, COSM580, COSM583, COSM584) and the wild-type allele. Each sample was then processed on the QX200 droplet digital PCR system (Bio-Rad), and data were manually analyzed with the QuantaSoft software (Bio-Rad) according to the manufacturer‘s instructions. Healthy controls were measured using the same method. ctDNA levels were calculated as the relative amount of mutated alleles over the total amount of molecules (mutated + wild-type) detected. 

To determine correlations of ctDNA levels with routine blood markers and tumor progression, only the first available ctDNA sample per patient was considered.

### 2.4. Disease Progression Assessment

FDG-PET-CT scans were performed at three-month intervals according to routine institutional procedures. We selected scans taken during the first 12 months after the initial plasma sample collection if follow-up data for 12 months were available. Tumor development was evaluated according to PET-CT response at the time point of the scan. To simplify the evaluation and data correlation, we dichotomized the tumor progression data compared to the last available image into two groups, namely “tumor control” and “tumor progression”. “Tumor control” comprised the assessments of metabolic complete response (MCR), metabolic partial response (MPR), and metabolic stable disease (MSD). “Tumor progression” included the assessment of metabolic progressive disease (MPD) and patients who died. In the event that death occurred before 12 months, patients were included in the “tumor progression” category to account for the missing time points (last observation carried forward (LOCF)). 

### 2.5. Statistics

Categorical variables were summarized as frequencies. To assess differences in categorical variables, chi-square and Fisher’s exact tests were used. Continuous variables were summarized using mean, median, and range. For continuous variables, a two-sided t-test was used. For univariate survival analysis, the log-rank test was utilized. For multivariate survival analysis, a Cox regression model was built.

Statistical analysis was performed with R, version 4.1.0 (R Foundation for statistical computing, Vienna, Austria, 2022). The significance level was determined at *p* < 0.05.

## 3. Results

### 3.1. Demographics

A total of 129 plasma samples from 79 patients were included in the analyses. Patient characteristics and clinical parameters are summarized in Table 1. The complete clinical data table has been added as a supplementary material (Table S1). 

### 3.2. M-Classification and Metastasis Location at Time of Sample Collection

**Figure 2:**
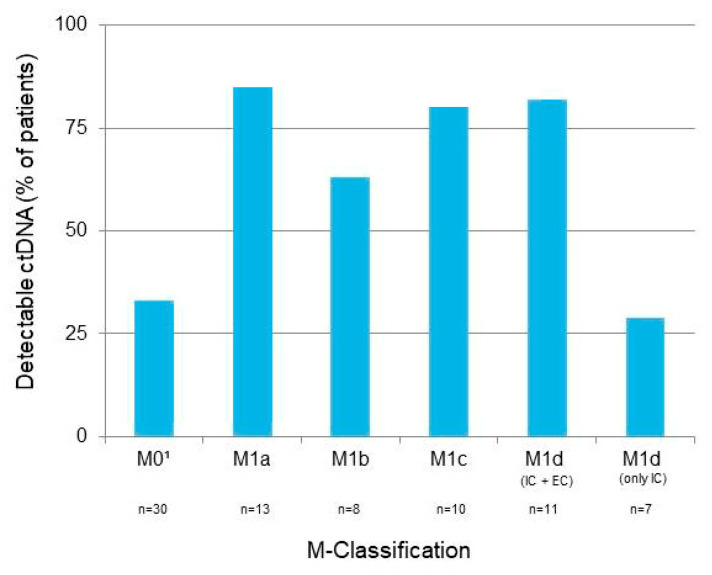
Metastasis staging according to the AJCC 8.0 M classification [17] at the time of the first ctDNA sample collection in patients with detectable and undetectable ctDNA. ^1^ M0, no distant metastasis; M1a, skin, soft tissue, and/or non-regional lymph nodes; M1b, pulmonary metastasis, with or without lesions from M1a; M1c, metastasis in other non-CNS visceral sites, with or without lesions from M1a and M1b; M1d, metastasis of the CNS, with (IC + EC) or without (IC only) M1a, M1b, and M1c.

In 49 out of 79 patients (62%), metastases were detected with a PET-CT scan at the time of the first ctDNA sample collection (Figure 2). ctDNA was detectable in a statistically significantly larger proportion of patients with distant metastases (79%; M1a, M1b, M1c, and M1d (IC + EC) than in patients with no distant metastases or only intracranial metastases (32%; M0 and M1d (only IC), *p* < 0.0001). 

### 3.3. Detectable ctDNA Correlates with Elevated S100 and CRP, but Not LDH

**Figure 3:**
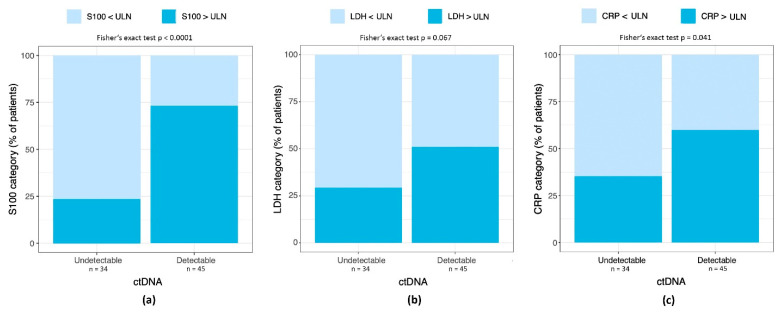
(**a**) Percentage of patients with S100 > ULN in patients with detectable and undetectable ctDNA. (**b**) Percentage of patients with LDH > ULN in patients with detectable and undetectable ctDNA. (**c**) Percentage of patients with CRP > ULN in patients with detectable and undetectable ctDNA.

We tested whether detectable ctDNA was associated with elevated S100, LDH, and CRP (Figure 3a–c). Patients with detectable ctDNA levels had a statistically significantly higher frequency of elevated S100 values (odds ratio (OR) = 5.16; 95% CI = 1.73 to 17.14; *p* ≤ 0.0001) and elevated CRP level (OR = 2.71; 95% CI = 1.00 to 7.69; *p* = 0.041). Elevations of LDH were numerically higher in the group with detectable ctDNA, but differences did not reach statistical significance (OR = 2.48; 95% CI = 0.89 to 7.25; *p* = 0.067). 

### 3.4. ctDNA as a Predictor for Tumor Progression and Overall Survival

#### 3.4.1. Percentage of Patients with Tumor Progression at 3, 6, 9, and 12 Months after ctDNA Sample

**Figure 4:**
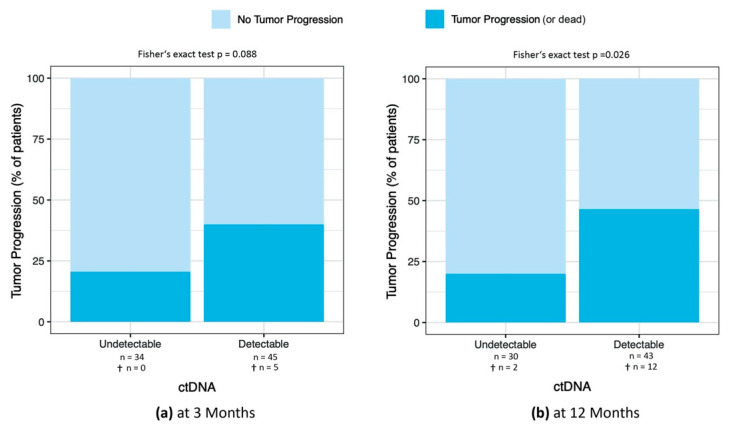
(**a**) Percentage of patients with tumor progression at 3 months after ctDNA sample collection in patients with detectable and undetectable ctDNA. (**b**) Percentage of patients with tumor progression at 12 months in patients with detectable and undetectable ctDNA. *p*-value was determined with Fisher’s exact test. At the time of data analysis, the follow-up results at 12 months for 6 patients were not yet available. †, number of patients who died.

To evaluate the predictive value of detectable ctDNA for tumor progression, we calculated the percentage of patients with tumor progression at 3, 6, 9, and 12 months after sample collection. At all four time points, a higher percentage of patients with detectable ctDNA at the time of the first sample showed tumor progression (at 3 months, HR = 2.54; 95% CI = 0.84 to 8.43; *p*-value = 0.088; at 6 months, HR = 2.01; 95% CI = 0.71 to 6.07; *p*-value = 0.16; at 9 months, HR = 2.75; 95% CI = 0.97 to 8.32; *p*-value = 0.039; at 12 months, HR = 2.90; 95% CI = 1.00 to 9.00; *p*-value = 0.026; results at 3 months and 12 months are shown in Figure 4, results at 6 and 9 months are shown in Appendix A). 

#### 3.4.2. ctDNA Detectability as a Predictor for Progression-Free Survival and Overall Survival

**Figure 5:**
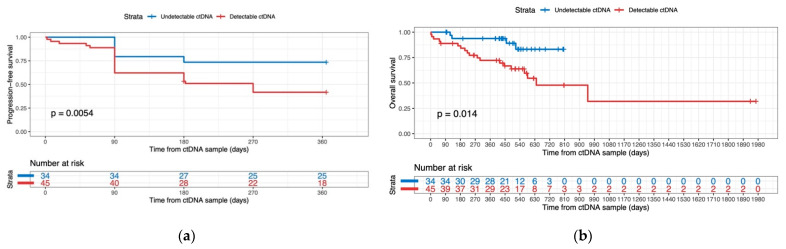
Comparison of detectable vs undetectable ctDNA at the time of the first plasma sample in regards to PFS (**a**) and OS (**b**). *p*-values were determined with log-rank tests.

We tested the associations between ctDNA detectability at the time point of the first plasma sample and PFS and OS (univariate analyses, Figure 5). 

To compare associations between detectable ctDNA, routine blood markers, known clinical prognostic factors, PFS, and OS, we calculated a multivariate Cox regression model (Table 2).

Univariate analyses showed direct associations between detectable ctDNA and significantly lower PFS (*p*-value = 0.0054) and OS (*p*-value = 0.014) rates. After adjustment for routine blood markers and prognostic clinical factors in multivariate analyses, the result was confirmed for OS (HR = 3.06; CI = 1.03 to 9.06; *p*-value = 0.044) but not PFS. 

### 3.5. Longitudinal Disease Monitoring with ctDNA

Of the 79 patients included, 15 patients had more than 2 ctDNA measurements. Of these, three patient examples with interesting ctDNA dynamics and the corresponding clinical course are illustrated in Appendix B. 

## 4. Discussion

We report a retrospective analysis of ctDNA measurements at non-predefined time points and their correlation with tumor progression and routine blood parameters in a cohort of patients with metastatic melanoma. Patients’ demographics in this study were representative of a population with metastatic melanoma stage IIIB–IV that is expected in a tertiary university hospital and is intended to reflect real-world circumstances.

The results of this study highlight the potential of ctDNA as a liquid biomarker to predict OS in patients with advanced melanoma, even if a single ctDNA measurement is collected at a random time point throughout the patient’s treatment. 

Our findings are in line with other published studies regarding the usefulness of ctDNA to monitor treatment success. Differences in the results can be explained by different study designs and patient populations [2,6,7,8,9,20]. Most of these were small retrospective studies with a short observation period and inconsistent sampling time points [8,9,20]. The only reported large prospective trial was a double-blind, randomized, therapeutic, phase 3, multicenter trial comparing dabrafenib plus trametinib versus dabrafenib plus placebo in previously untreated patients with metastatic melanoma. A total of 423 patients were included in this study. A subgroup analysis showed that the quantity of ctDNA at baseline correlated negatively with PFS and OS [7].

However, the conclusions of published clinical studies are equivocal regarding the usefulness of ctDNA in the routine clinical practice of patients with advanced melanoma. There is a lack of clinical validation for the majority of ctDNA assays [22]. Plasma levels of ctDNA are influenced by various factors, most of which are largely unknown [5,23,24,25,26,27,28,29].

Furthermore, this study supports the findings of other studies that ctDNA measured in plasma has only limited value in monitoring intracranial disease activity [7,20,30,31].

The assessment of ctDNA has the potential to improve the clinical management of patients with metastatic melanoma [7,8,9,20,32]. However, there are important aspects that require caution in the interpretation of ctDNA assessments to exploit its full range of potential benefits: (1) Changes in ctDNA concentration during therapy can provide valuable information regarding treatment response and its duration. Furthermore, an increase in ctDNA concentration after an initial decrease can suggest a loss of initial response to the therapeutic agent and may trigger a switch of therapy. (2) The number of ctDNA copies per mL appears to be inversely associated with treatment response, progression-free survival, and overall survival [7,8]. Quantitative ctDNA measurements have the potential to improve the predictive value of this marker significantly. (3) Handling of blood samples and storage until analysis should be standardized. (4) A serious limitation of plasma ctDNA is that brain melanoma metastases and their dynamics are not reflected in changes in this biomarker. The measurement of ctDNA concentration in CSF may represent an alternative, but only limited data on its usefulness are available. Imaging methods remain, for the time being, the gold standard for the detection and monitoring of brain metastases [7,20,30,31]. 

This study found that patients with only intracranial disease showed a lower detectability of ctDNA, underlining the need for further development of biomarkers. Additionally, the detectability of ctDNA correlated with elevated S100 and CRP. Larger prospective studies are needed to investigate at which time points and at which frequency ctDNA should be measured to monitor treatment responses more accurately.

## 5. Conclusions

The application of ctDNA as a liquid biomarker in the management of patients with metastatic melanoma is promising. Its potential can best be exploited when it will be assessed quantitatively as early as possible after diagnosis and repeated at regular time intervals. This study shows that even ctDNA measurements, which were taken at random time points, may have a prognostic value. ctDNA allows for the monitoring of therapy and the detection of disease progression probably earlier than any other method. Further development of biomarkers is needed for the identification and monitoring of brain metastases, as the value of ctDNA measured in plasma seems to be limited. Large prospective, longitudinal clinical studies are needed to clarify its definitive role in the management of patients with malignant melanoma.

## Figures and Tables

**Table 1:** Patients’ characteristics in the overall population, in patients with detectable ctDNA and undetectable ctDNA.

Characteristic	Total n = 79	Detectable ctDNA n = 45 (57%)	Undetectable ctDNA n = 34 (43%)	*p*-Value ^1^
**Age (years)**				0.086
Mean (±SD)	63.3 (±14.3)	65.8 (±13.4)	60.1 (±14.9)	
Median (Min., Max.)	62.0 (24, 88)	67.0 (31, 88)	59.5 (24, 87)	
**Gender**				0.831
Female	28 (35%)	15 (33%)	13 (38%)	
Male	51 (65%)	30 (67%)	21 (62%)	
**Tumor Stage**				0.308
IIIB	6 (8%)	2 (4%)	4 (12%)	
IIIC	9 (11%)	4 (9%)	5 (15%)	
IV	64 (81%)	39 (87%)	25 (73%)	
**Breslow Thickness**				0.818
Mean (±SD)	3.08 (±2.97)	2.99 (±2.08)	3.17 (±3.75)	
Median (Min., Max.)	2.10 (0.6, 20.0)	2.66 (0.6, 9.0)	1.92 (0.6, 20.0)	
Missing	18 (23%)	13 (29%)	5 (15%)	
**Ulceration**				0.668
No	42 (53%)	21 (47%)	21 (62%)	
Yes	22 (28%)	13 (29%)	9 (26%)	
Missing	15 (19%)	11 (24%)	4 (12%)	
**Driver Mutation**				0.843
BRAF p.V600E	43 (55%)	25 (56%)	18 (53%)	
BRAF p.V600K	9 (11%)	5 (11%)	4 (11%)	
NRAS p.Q61K	16 (20%)	10 (22%)	6 (18%)	
NRAS p.Q61R	11 (14%)	5 (11%)	6 (18%)	
**Therapy at Sampling**				0.184
Targeted therapy ^2^	14 (18%)	9 (20%)	5 (15%)	
Immunotherapy ^3^	43 (54%)	20 (44%)	23 (67%)	
Chemotherapy	3 (4%)	3 (7%)	0	
Local therapy ^4^	15 (19%)	11 (25%)	4 (12%)	
None	4 (5%)	2 (4%)	2 (6%)	

^1^ Two-sided t-test; chi-square test; ^2^ MAPK inhibitors, individual agents or in combinations; ^3^ anti-PD1, anti-CTLA4, anti-LAG3, individual agents or in combinations; ^4^ surgery or radiotherapy.

**Table 2:** Multivariate Cox model for progression-free and overall survival, including ctDNA detection, routine blood markers ^1^, and clinical factors ^2^.

	Coef	HR (95% CI)	SE (coef)	z	*p*-Value
**Progression-free survival**					
Female Gender	−0.87	0.42 (0.20–0.88)	0.38	−2.30	0.021
S100 > ULN	1.55	4.74 (2.18–10.27)	0.39	3.94	<0.0001
CRP > ULN	1.18	3.27 (1.49–7.18)	0.40	2.95	0.003
Presence of Intracranial Metastases	0.97	2.65 (1.32–5.32)	0.36	2.74	0.006
**Overall Survival**					
Detectable ctDNA	1.17	3.06 (1.03–9.06)	0.55	2.02	0.044
CRP > ULN	1.65	4.36 (1.71–12.07)	0.62	2.19	0.013

Analysis includes 79 patients; ^1^ LDH > ULN, S100 > ULN, and CRP > ULN; ^2^ Breslow thickness and ulceration of primary tumor, age, gender, therapies at the time of sampling, and location of metastases; coef, coefficient; HR, hazard ratio; SE, standard error. ULN, upper limit of normal.

**Figure A1:**
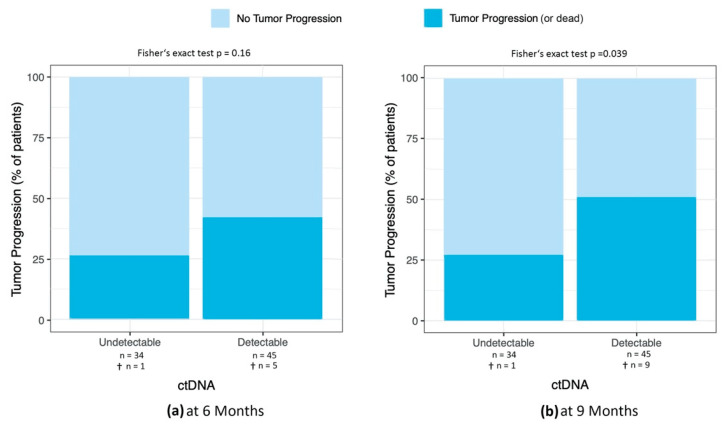
(**a**) Percentage of patients with tumor progression at 6 months after ctDNA sample collection in patients with detectable and undetectable ctDNA. (**b**) Percentage of patients with tumor progression at 9 months in patients with detectable and undetectable ctDNA. The *p*-value was determined using Fisher’s exact test. †: Number of patients who died.

**Figure A2:**
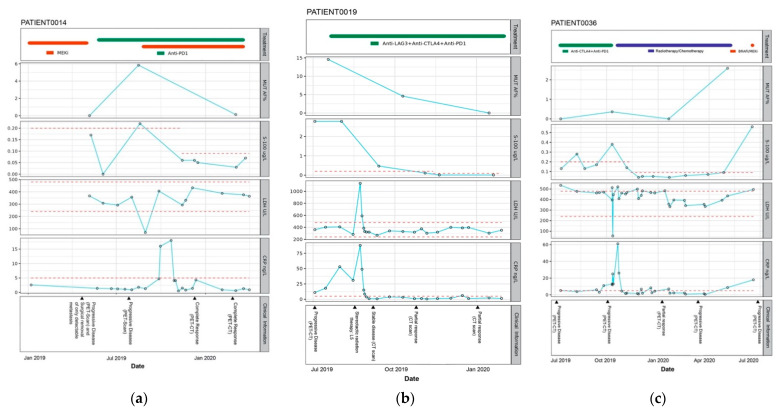
Longitudinal measurements of ctDNA, S100, LDH, CRP, Therapeutic Interventions, and PET-CT results over time in three patient examples. Dashed red lines: S100: ULN (Assay changed in December 2019, different ULN); LDH, ULN and 2× ULN; CRP, ULN; Mut AF; Mutant Allele Frequency.

## Data Availability

An excel table with all clinical data and ctDNA measurements has been added as a Supplementary Material.

## Supplementary Materials

The excel data table, consisting of clinical data and ctDNA measurements, can be downloaded at https://www.mdpi.com/article/10.3390/cancers14174158/s1, Table S1. ctDNA Data Table.

## Appendix B

### Longitudinal Disease Monitoring with ctDNA

Three patient examples with multiple ctDNA and routine blood marker measurements are individually shown in Figure A2.

Patient 0014 (Figure A2a): This patient with stage IV melanoma (M1a, *NRAS* Q61K mutated, COSM580) received MEKi monotherapy from March 2015–May 2019. In May 2019, the patient developed a solitary detectable metastasis, which was surgically removed. ctDNA could not be detected in the first assessment shortly after surgery. Thereafter, adjuvant immunotherapy with an anti-PD1 antibody was started. In August 2019, the patient developed a relapse. At this time point, ctDNA was detectable. After that, the patient started a combined treatment with an anti-PD1 antibody + MEKi and reached a complete response in December 2019. In January 2020, ctDNA returned to undetectable levels. S100 showed a similar pattern to ctDNA measurements. LDH ranged between ULN and 2x ULN at all time points, with the exception of one measurement in August 2019, which was 30 U/L, which may represent a measurement error. CRP showed elevated levels after the re-initiation of MEKi, which could be related to tumor necrosis.

Patient 0019 (Figure A2b): This stage IV (M1c, *BRAF* V600E mutated, COSM476) melanoma patient responded well to the combination treatment consisting of anti-LAG3 + anti-CTLA4, and anti-PD1 immunotherapy. ctDNA and S100 showed a similar time course. In August 2019, local radiotherapy of a bone metastasis located in L5 was performed. Consequently, LDH and CRP showed a peak in concentration.

Patient 0036 (Figure A2c): This patient with intra- and extracranial disease (stage IV (M1d), *BRAF* V600K mutated, COSM473) responded poorly to different treatment modalities. After three months of immunotherapy with anti-CTLA4 + anti-PD1, tumor progression was identified in a PET-CT scan, and as a consequence, therapy was switched to chemotherapy and radiotherapy for cerebral metastases. Under this treatment, the patient reached a short-term partial response after three months but showed tumor progression again after six months in April 2020. ctDNA levels mimicked a therapeutic response, S100 did so only partially, without elevated concentration, in April 2020. LDH was slightly elevated throughout the whole observation period, with the exception of one low value in October 2019, which may represent a measurement error with no clinical correlation. CRP peaked after therapy initiation with chemo- and radiotherapy in October 2019.

In summary, these patients with different disease courses show concomitant elevation of ctDNA and S100 with tumor progression, whereas LDH and CRP were less specific.

## References

[1] Gray E.S., Rizos H., Reid A.L., Boyd S.C., Pereira M.R., Lo J., Tembe V., Freeman J., Lee J.H., Scolyer R.A. (2015). Circulating tumor DNA to monitor treatment response and detect acquired resistance in patients with metastatic melanoma. Oncotarget.

[2] Schreuer M., Meersseman G., Van Den Herrewegen S., Jansen Y., Chevolet I., Bott A., Wilgenhof S., Seremet T., Jacobs B., Buyl R. (2016). Quantitative assessment of BRAF V600 mutant circulating cell-free tumor DNA as a tool for therapeutic monitoring in metastatic melanoma patients treated with BRAF/MEK inhibitors. J. Transl. Med..

[3] Wan J.C.M., Massie C., Garcia-Corbacho J., Mouliere F., Brenton J.D., Caldas C., Pacey S., Baird R., Rosenfeld N. (2017). Liquid biopsies come of age: Towards implementation of circulating tumour DNA. Nat. Rev. Cancer.

[4] Pessoa L.S., Heringer M., Ferrer V.P. (2020). ctDNA as a cancer biomarker: A broad overview. Crit. Rev. Oncol. Hematol..

[5] Bettegowda C., Sausen M., Leary R.J., Kinde I., Wang Y., Agrawal N., Bartlett B.R., Wang H., Luber B., Alani R.M. (2014). Detection of circulating tumor DNA in early- and late-stage human malignancies. Sci. Transl. Med..

[6] Gracie L., Pan Y., Atenafu E.G., Ward D.G., Teng M., Pallan L., Stevens N.M., Khoja L. (2021). Circulating tumour DNA (ctDNA) in metastatic melanoma, a systematic review and meta-analysis. Eur. J. Cancer.

[7] Syeda M.M., Wiggins J.M., Corless B.C., Long G.V., Flaherty K.T., Schadendorf D., Nathan P.D., Robert C., Ribas A., Davies M.A. (2021). Circulating tumour DNA in patients with advanced melanoma treated with dabrafenib or dabrafenib plus trametinib: A clinical validation study. Lancet Oncol..

[8] Váraljai R., Wistuba-Hamprecht K., Seremet T., Diaz J.M.S., Nsengimana J., Sucker A., Griewank K., Placke J.M., Horn P.A., von Neuhoff N. (2020). Application of Circulating Cell-Free Tumor DNA Profiles for Therapeutic Monitoring and Outcome Prediction in Genetically Heterogeneous Metastatic Melanoma. JCO Precis. Oncol..

[9] Knuever J., Weiss J., Persa O.D., Kreuzer K., Mauch C., Hallek M., Schlaak M. (2020). The use of circulating cell-free tumor DNA in routine diagnostics of metastatic melanoma patients. Sci. Rep..

[10] McEvoy A.C., Pereira M.R., Reid A., Pearce R., Cowell L., Al-Ogaili Z., Khattak M.A., Millward M., Meniawy T.M., Gray E.S. (2019). Monitoring melanoma recurrence with circulating tumor DNA: A proof of concept from three case studies. Oncotarget.

[11] Garlan F., Blanchet B., Kramkimel N., Puszkiel A., Golmard J.L., Noe G., Dupin N., Laurent-Puig P., Vidal M., Taly V. (2017). Circulating Tumor DNA Measurement by Picoliter Droplet-Based Digital PCR and Vemurafenib Plasma Concentrations in Patients with Advanced BRAF-Mutated Melanoma. Target Oncol..

[12] Braune J., Keller L., Schiller F., Graf E., Rafei-Shamsabadi D., Wehrle J., Follo M., Philipp U., Hussung S., Pfeifer D. (2020). Circulating Tumor DNA Allows Early Treatment Monitoring in BRAF- and NRAS-Mutant Malignant Melanoma. JCO Precis. Oncol..

[13] Forthun R.B., Hovland R., Schuster C., Puntervoll H., Brodal H.P., Namløs H.M., Aasheim L.B., Meza-Zepeda L.A., Gjertsen B.T., Knappskog S. (2019). ctDNA detected by ddPCR reveals changes in tumour load in metastatic malignant melanoma treated with bevacizumab. Sci. Rep..

[14] McEvoy A.C., Warburton L., Al-Ogaili Z., Celliers L., Calapre L., Pereira M.R., Khattak M.A., Meniawy T.M., Millward M., Ziman M. (2018). Correlation between circulating tumour DNA and metabolic tumour burden in metastatic melanoma patients. BMC Cancer.

[15] Tolmeijer S.H., Koornstra R.H.T., de Groot J.W.B., Geerlings M.J., van Rens D.H., Boers-Sonderen M.J., Schalken J.A., Gerritsen W.R., Ligtenberg M.J.L., Mehra N. (2021). Plasma BRAF Mutation Detection for the Diagnostic and Monitoring Trajectory of Patients with LDH-High Stage IV Melanoma. Cancers.

[16] Seremet T., Jansen Y., Planken S., Njimi H., Delaunoy M., El Housni H., Awada G., Schwarze J.K., Keyaerts M., Everaert H. (2019). Undetectable circulating tumor DNA (ctDNA) levels correlate with favorable outcome in metastatic melanoma patients treated with anti-PD1 therapy. J. Transl. Med..

[17] Amin M.B., Greene F.L., Edge S.B., Compton C.C., Gershenwald J.E., Brookland R.K., Meyer L., Gress D.M., Byrd D.R., Winchester D.P. (2017). The Eighth Edition AJCC Cancer Staging Manual: Continuing to build a bridge from a population-based to a more “personalized” approach to cancer staging. CA Cancer J. Clin..

[18] Gaynor R., Herschman H.R., Irie R., Jones P., Morton D., Cochran A. (1981). S100 protein: A marker for human malignant melanomas?. Lancet.

[19] Henze G., Dummer R., Joller-Jemelka H.I., Böni R., Burg G. (1997). Serum S100—A marker for disease monitoring in metastatic melanoma. Dermatology.

[20] Marsavela G., McEvoy A.C., Pereira M.R., Reid A.L., Al-Ogaili Z., Warburton L., Khattak M.A., Abed A., Meniawy T.M., Millward M. (2022). Detection of clinical progression through plasma ctDNA in metastatic melanoma patients: A comparison to radiological progression. Br. J. Cancer.

[21] Griewank K.G., Schilling B. (2017). Next-Generation Sequencing to Guide Treatment of Advanced Melanoma. Am. J. Clin. Dermatol..

[22] Merker J.D., Oxnard G.R., Compton C., Diehn M., Hurley P., Lazar A.J., Lindeman N., Lockwood C.M., Rai A.J., Schilsky R.L. (2018). Circulating Tumor DNA Analysis in Patients With Cancer: American Society of Clinical Oncology and College of American Pathologists Joint Review. J. Clin. Oncol..

[23] Schwarzenbach H., Hoon D.S., Pantel K. (2011). Cell-free nucleic acids as biomarkers in cancer patients. Nat. Rev. Cancer.

[24] Kustanovich A., Schwartz R., Peretz T., Grinshpun A. (2019). Life and death of circulating cell-free DNA. Cancer Biol. Ther..

[25] Peters D.L., Pretorius P.J. (2011). Origin, translocation and destination of extracellular occurring DNA—A new paradigm in genetic behaviour. Clin. Chim. Acta.

[26] Muhanna N., Di Grappa M.A., Chan H.H.L., Khan T., Jin C.S., Zheng Y., Irish J.C., Bratman S.V. (2017). Cell-Free DNA Kinetics in a Pre-Clinical Model of Head and Neck Cancer. Sci. Rep..

[27] Muhanna N., Eu D., Chan H.H.L., Douglas C., Townson J.L., Di Grappa M.A., Mohamadi R.M., Kelley S.O., Bratman S.V., Irish J.C. (2021). Cell-free DNA and circulating tumor cell kinetics in a pre-clinical head and neck Cancer model undergoing radiation therapy. BMC Cancer.

[28] Diehl F., Schmidt K., Choti M.A., Romans K., Goodman S., Li M., Thornton K., Agrawal N., Sokoll L., Szabo S.A. (2008). Circulating mutant DNA to assess tumor dynamics. Nat. Med..

[29] Leung F., Kulasingam V., Diamandis E.P., Hoon D.S., Kinzler K., Pantel K., Alix-Panabieres C. (2016). Circulating Tumor DNA as a Cancer Biomarker: Fact or Fiction?. Clin. Chem..

[30] Davies M.A., Liu P., McIntyre S., Kim K.B., Papadopoulos N., Hwu W.J., Hwu P., Bedikian A. (2011). Prognostic factors for survival in melanoma patients with brain metastases. Cancer.

[31] Lee J.H., Menzies A.M., Carlino M.S., McEvoy A.C., Sandhu S., Weppler A.M., Diefenbach R.J., Dawson S.J., Kefford R.F., Millward M.J. (2020). Longitudinal Monitoring of ctDNA in Patients with Melanoma and Brain Metastases Treated with Immune Checkpoint Inhibitors. Clin. Cancer Res..

[32] Siravegna G., Mussolin B., Venesio T., Marsoni S., Seoane J., Dive C., Papadopoulos N., Kopetz S., Corcoran R.B., Siu L.L. (2019). How liquid biopsies can change clinical practice in oncology. Ann. Oncol..

